# The role of regenerative therapy in the treatment of right ventricular failure: a literature review

**DOI:** 10.1186/s13287-020-02022-w

**Published:** 2020-11-25

**Authors:** Christoph Haller, Mark K. Friedberg, Michael A. Laflamme

**Affiliations:** 1 Division of Cardiovascular Surgery, The Labatt Family Heart Centre, The Hospital for Sick Children, Toronto, Canada; 2 Department of Surgery, University of Toronto, Toronto, Canada; 3 McEwen Stem Cell Institute, Peter Munk Cardiac Centre, University Health Network, Toronto, Canada; 4 Division of Cardiology, The Labatt Family Heart Centre, The Hospital for Sick Children, Toronto, Canada; 5 Department of Pediatrics, University of Toronto, Toronto, Canada; 6 Department of Physiology, University of Toronto, Toronto, Canada; 7 Department of Laboratory Medicine and Pathobiology, University of Toronto, Toronto, Canada; 8 McEwen Stem Cell Institute, Toronto Medical Discovery Tower, 101 College Street, Toronto, Ontario M5G 1L7 Canada

**Keywords:** Cardiac regeneration, Pluripotent stem cells, Right ventricle, Heart failure, Congenital heart disease, Pulmonary hypertension

## Abstract

Right ventricular (RV) failure is a commonly encountered problem in patients with congenital heart disease but can also be a consequence of left ventricular disease, primary pulmonary hypertension, or RV-specific cardiomyopathies. Improved survival of the aforementioned pathologies has led to increasing numbers of patients suffering from RV dysfunction, making it a key contributor to morbidity and mortality in this population. Currently available therapies for heart failure were developed for the left ventricle (LV), and there is clear evidence that LV-specific strategies are insufficient or inadequate for the RV. New therapeutic strategies are needed to address this growing clinical problem, and stem cells show significant promise. However, to properly evaluate the prospects of a potential stem cell-based therapy for RV failure, one needs to understand the unique pathophysiology of RV dysfunction and carefully consider available data from animal models and human clinical trials. In this review, we provide a comprehensive overview of the molecular mechanisms involved in RV failure such as hypertrophy, fibrosis, inflammation, changes in energy metabolism, calcium handling, decreasing RV contractility, and apoptosis. We also summarize the available preclinical and clinical experience with RV-specific stem cell therapies, covering the broad spectrum of stem cell sources used to date. We describe two different scientific rationales for stem cell transplantation, one of which seeks to add contractile units to the failing myocardium, while the other aims to augment endogenous repair mechanisms and/or attenuate harmful remodeling. We emphasize the limitations and challenges of regenerative strategies, but also highlight the characteristics of the failing RV myocardium that make it a promising target for stem cell therapy.

## Background

Right ventricular (RV) failure is an important but often overlooked cause of heart failure. Historically, the importance of the RV has been underestimated because the pulmonary vasculature can be perfused passively [1]. However, it is now clear that RV dysfunction in various conditions is a significant risk factor for poor outcome, it is highly prevalent, and the population at risk for RV failure is increasing [2]. RV failure can result from several etiologies, ranging from failure secondary to left ventricular (LV) disease, pulmonary hypertension of various causes, and RV-specific cardiomyopathies to sequelae of uncorrected, palliated, or corrected congenital heart disease (CHD). CHD affects approximately 1% of live births worldwide. Outcomes have significantly improved; but morbidity remains high, and children with CHD are more likely to suffer from worse health, additional medical conditions, and to consume more healthcare [3]. Right-sided heart failure is a frequent underlying cause and importantly impacts adverse outcomes in the CHD population. Projected costs of heart failure are expected to more than double, reaching $69.7 billion in 2030 [4]. Pediatric heart failure is an important contributor due to higher rates of mechanical ventilation, heart transplantation, and surgical intervention compared to adult patients with heart failure [5]. Furthermore, CHD patients surviving to adulthood now outnumber pediatric CHD patients, further increasing the population at risk for RV failure.

A common mechanism of RV failure is its exposure to high, non-physiologic pressures and/or volume overload. RV pressure overload causes reactive hypertrophy, capillary rarefaction, and generation of oxidative stress, which ultimately leads to fibrosis, cardiomyocyte dysfunction, and cardiomyocyte loss [6]. Therapeutic strategies are limited, especially as basic and clinical research has historically focused mainly on the LV. Furthermore, pediatric myocardium responds to pharmacological treatments differently than in adults, and therapies effective in LV failure have been proven ineffective or even harmful in RV failure [7]. Surgical or catheter-based interventions often provide inadequate or temporary relief of pressure or volume overload and cannot prevent RV failure. It is therefore essential to develop new therapeutic strategies to prevent or reverse RV failure, and regenerative and/or cell-based approaches have engendered considerable interest. In this article, we review the unique pathophysiology of RV failure and summarize data on cell-based strategies from preclinical studies and early-phase human trials.

## Main text

### Right ventricular pathophysiology

The principal mechanisms that lead to RV dysfunction and failure are increased RV pressure and/or volume loading, or some combination of these two conditions. Volume loading, caused by intra- or extracardiac shunts and valve regurgitation, is commonly seen in CHD patients. Pressure loading and increased pulmonary vascular resistance are more common causes of RV dysfunction, resulting from uncorrected CHD, from a consequence of CHD surgical repair, or from pulmonary hypertension, for example secondary to LV failure. Here, prolonged exposure to high afterload ultimately increases the risk for low RV output and death [8].

There are fundamental morphologic differences between the RV and the LV that correspond to their respective functions within the cardiovascular system. As opposed to the heavily trabeculated right chamber, the cone-shaped LV myocardium is compact and thick. Fiber orientation in the LV is predominantly concentric, and its layered architecture is optimized for the generation of high pressures via radial contraction. By contrast, the RV has longitudinally oriented fibers that are better suited to eject volume into the normally low impedance pulmonary circulation. The RV typically faces significantly lower afterload than the LV and has lower hydraulic impedance and higher compliance. Normally, the RV needs only a fifth of LV energy consumption to maintain the same cardiac output [9]. In pulmonary hypertension, RV pressure overload can result in a fivefold increase in afterload as compared to the 1.5-fold increase in LV afterload typical in systemic hypertension [10]. In hypoplastic left heart syndrome (HLHS), a congenital defect in which the LV is underdeveloped and the RV supplies the systemic circulation, the RV has to generate up to 280% of normal cardiac output at much higher pressure [11]. Cardiac output may drop disproportionately with changes in afterload. Moreover, RV contraction is often inefficient with regional heterogeneity and dyssynchronous contraction [12]. The ensuing transition from adaptive to maladaptive hypertrophy plays a critical role in chronic RV failure. The RV’s initial response is adaptive hypertrophy with preserved cardiac output and little fibrosis or dilatation [13]. Mechanical stress is a key factor activating maladaptive responses including fibrosis, capillary rarefaction, oxidative stress, cardiomyocyte dysfunction, and decreasing numbers of functioning cardiomyocytes [6, 10]. These ultimately are associated with RV remodeling and dilatation which are powerful predictors of death or need for transplantation [14].

RV pressure overload is associated with ischemia caused by reduced right coronary perfusion and capillary rarefaction [13]. As a result, there is an early increase in the disease process in mitochondrial reactive oxygen species (ROS), leading to hypoxia-inducible factor 1α (HIF1α) inhibition and p53 activation [15]. HIF1α inhibition further reduces angiogenesis. In contrast to the LV, the effects of HIF1α/vascular endothelial growth factor (VEGF) signaling differ depending on the etiology of increased pulmonary afterload. The RV does not typically respond with increased myocardial capillary density, and this situation may contribute to metabolic changes and fibrosis [6]. Angiogenic factors such as VEGF, angiopoietin-1, and apelin are downregulated, likely contributing to impaired capillary growth and maintenance [16]. Furthermore, transforming growth factor β1 (TGF-β1), connective tissue growth factor (CTGF), endothelin-1, endothelin receptor B, and matrix metalloproteinase 2/9 mRNA levels are increased [17]. Endothelin-1 and the TGF-β1–endothelin-1–CTGF axis are potent inductors of extra-cellular matrix remodeling and fibrosis. Interestingly, myocardial fibrosis does not occur uniformly throughout the RV in pressure overload but is instead emphasized at the septal hinge-points [18]. Inhibition of endothelin-1 ameliorates the pro-fibrotic response and may therefore exert a beneficial effect beyond pulmonary vascular dilation alone [19]. ROS also lead to altered expression of cMyc and forkhead box protein O1 (FOXO1), thereby influencing pyruvate dehydrogenase kinase (PDK) and oxidative metabolism [20]. Oxidative stress also induces an increase in cytosolic Ca^2+^ release by ryanodine receptor 2 activation and sarcoplasmic reticulum Ca^2+^-ATPase inhibition, further exacerbating RV dysfunction [20]. Ischemia also leads to a direct loss of cardiomyocytes [10].

As outlined above, pressure overload leads to activation of oxidative stress-induced transcription factors. The switch from adaptive to maladaptive hypertrophy is accompanied by activation of fetal genes such as atrial and brain natriuretic peptide, skeletal α-actin and β-myosin heavy chain (MHC), hyperpolarization-activated cyclic nucleotide-gated channel, and T-type Ca^2+^ channel as well as smooth muscle α-actin and SM22α [21]. Because the embryologic origin of the RV differs from the LV, its transcriptional response to increased afterload differs from that in the LV. Ovine models have shown that RV pressure overload leads to reactivation of the fetal gene program with increased cardiac expression of myocyte enhancer factor-2 (MEF-2), GATA-4, Nkx2.5, transcriptional enhancer factor 1 (TEF-1), and specificity protein 1 (Sp1, [22]). The α-myosin heavy chain (MHC) isoform predominates in the RV myocardium. However, during maladaptive hypertrophy, there is reduced α-MHC and increased β-MHC expression accompanied by decreased RV contractility [23].

**Fig. 1:**
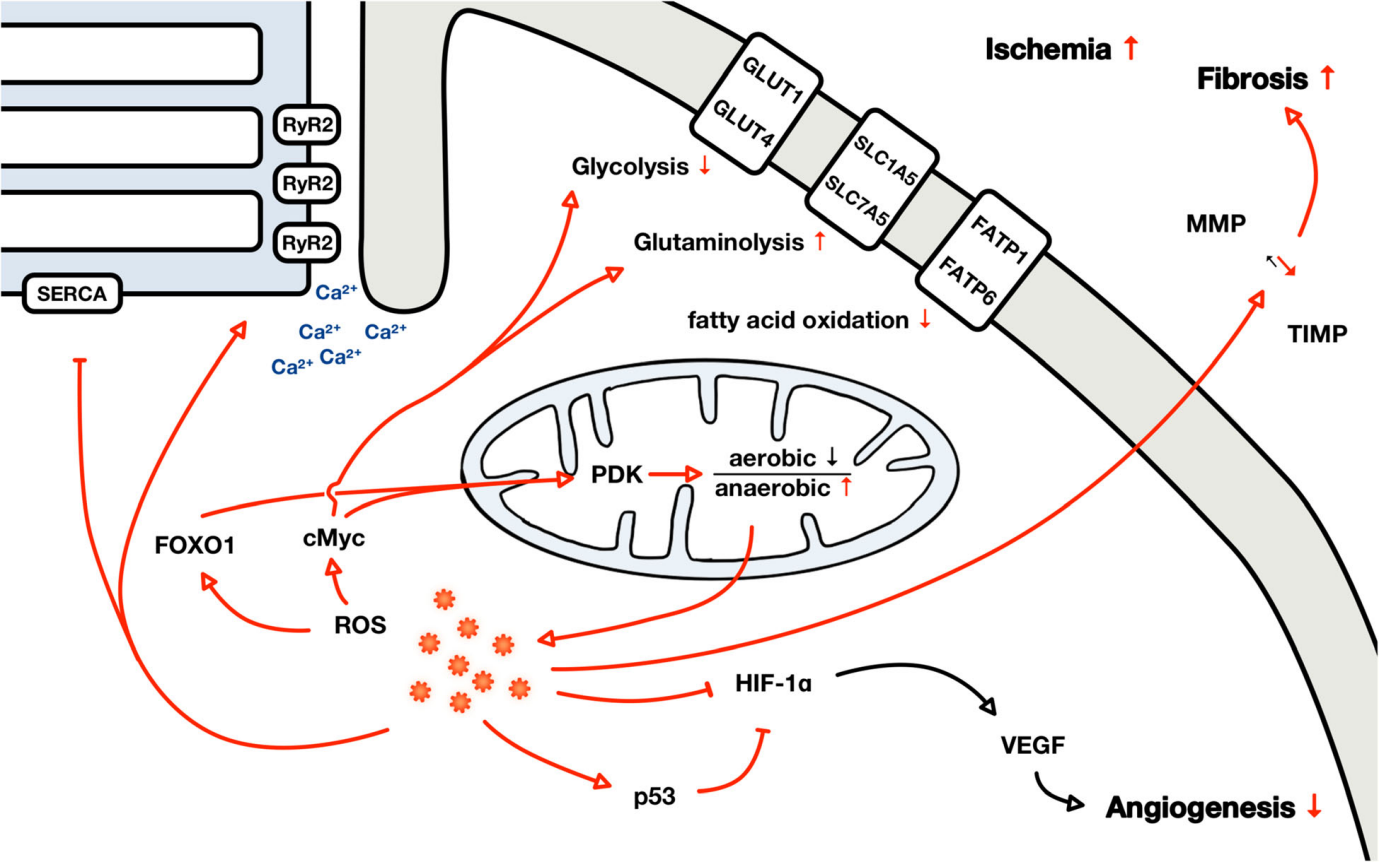
During RV pressure overload, elevated transmural wall pressures and reduced coronary perfusion results in ischemia and, in turn, increased production of reactive oxygen species (ROS). The ensuing direct and indirect inhibition of HIF1α leads to reduced angiogenesis via the downregulation of the VEGF axis. Imbalances between matrix metalloproteinase and tissue inhibitor metalloproteinases result in myocardial fibrosis. Alterations in pyruvate dehydrogenase kinase-related signaling drive a shift towards increased anaerobic glycolysis and glutaminolysis, further aggravating oxidative stress. Inhibited intracellular calcium release from sarcoplasmic reticulum stores via perturbed RyR2 and SERCA function contribute to contractile dysfunction in RV cardiomyocytes

Increased afterload also induces metabolic changes in the RV. During adaptive RV hypertrophy, glycolysis predominates with reduced fatty acid oxidation. The resultant situation resembles conditions of low fatty acid oxidation in the fetal heart. The aforementioned upregulation of FOXO1, c-Myc, and HIF1α, triggered by RV ischemia and ROS generation, activates PDK2 and 4, the predominant isoforms in the heart, and further shifts RV metabolism towards glycolysis and upregulation of glucose uptake [6, 20]. Glucose uptake is increased by the upregulation of glucose transporter 1 (Glut1). This metabolic shift is initially beneficial, as it reduces ROS and increases angiogenesis via the HIF1α/VEGF axis [15, 24]. c-Myc activation increases glutaminolysis, which is characteristic of cancer metabolism [25]. In the transition from adaptive to maladaptive RV hypertrophy, ATP is increasingly generated by anaerobic glycolysis and glutaminolysis. Increasing levels of ROS inhibit HIF1α and activate p53. This ultimately leads to a reversal in the metabolic profile, and the RV enters a positive feedback loop of p53 upregulation and HIF1α downregulation that hinders RV recovery [15]. The molecular pathways underlying this pathogenesis are depicted in Fig. 1.

Inflammation is a less well understood contributor to RV failure. The pathology of RV failure discussed above, such as RV hypertrophy, fibrosis, changes in energy metabolism, apoptosis, changes in calcium handling, and decreasing RV contractility, can increase inflammatory processes in the RV. On the other hand, inflammation at the myocardial level as well as indirect effects through circulating pro-inflammatory mediators can trigger or aggravate the very same array of pathophysiologic responses, leading into a vicious circle of negative effects. An in-depth review of inflammation in RV failure has been published by Dewachter et al. [26].

In summary, RV pressure overload elicits a characteristic response that leads to contractile dysfunction, fibrosis, altered gene expression, and metabolic changes. Lack of contractile force is a key underlying element, and myocyte hypertrophy is a compensatory response that initially serves to preserve cardiac output. With ongoing exposure to high afterload, however, hypertrophy alone is insufficient, and the mechanical stress and relative hypoxia activate pro-fibrotic, as well as pro-inflammatory pathways, and maladaptive remodeling.

### Stem cell therapy and its potential role in RV failure

Stem cells represent a relatively new and promising approach to heart failure in general, and multiple cell types have been explored in both animal models and early clinical trials. Importantly, these efforts have been motivated by two very different scientific rationales that have major implications with regard to the most appropriate cell type, dose, timing, and route of administration. First, some candidate cell therapies seek to “buttress” the myocardium via the direct regeneration or repopulation of the myocardium with new force-generating units. Other candidate cell therapies appear to function via indirect mechanisms, e.g., by the paracrine stimulation of intrinsic reparative responses and attenuation of harmful remodeling processes.

Most published studies in the stem cell field have examined outcomes in either animal models of LV myocardial infarction or adult patients with ischemic heart disease. However, more recent preclinical and early human studies have focused on examining the potential therapeutic efficacy of stem cells in RV-centric disease and pediatric patients [27–29]. Most of our knowledge regarding the role of stem cells in the treatment of RV failure has been gleaned from studies in various experimental animal models of increased RV afterload, such as pulmonary artery banding (PAB), monocrotaline, or Sugen 5416/hypoxia models. To date, bone marrow-derived mononuclear cells [30–36], umbilical cord blood-derived mononuclear cells [37–40], cardiosphere-derived cells, and cardiac progenitor cells [27, 28, 41–44] have been tested in animal models as well as in human trials to address RV failure (Tables 1 and 2). Beneficial effects have been reported following administration of each of these cell types, but preclinical studies have lacked uniform experimental design (i.e., have varied route of administration, number of cells delivered, animal models or disease context, etc.), and the currently available clinical data is limited by very small patient numbers. The few available meta-analyses have yielded variable results with regard to effects on myocardial function [46, 47]. There is also tremendous mechanistic uncertainty, and it has been variously suggested that tested cell therapies act either by direct regeneration of myocardium or via paracrine secretion of factors that trigger endogenous reparative mechanisms [30, 48].

**Table 1:** Preclinical cell-based studies in RV pressure-overload models

	Route of administration	Dose	Model	Species	Outcome
**BM-derived MSCs**					
Wehman et al. [32]	Intramyocardial	1.0 × 10^6^	PAB	Swine	RVfx ↑, angiogenesis ↑, hypertrophy ↓
Liufu et al. [31]	Intramyocardial	1.0 × 10^6^	PAB	Mouse	RV dimension ↓, hypertrophy ↓
**UCB-derived MNCs**					
Oommen et al. [37]	Intramyocardial	0.4 × 10^6^	PAB	Mouse	RVfx ↑, fibrosis ↓, angiogenesis ↑, pathogenic genes ↓
Davies et al. [38]	Epicardial	4.7 × 10^6^	PAB	Sheep	RV compliance ↑, recruitable stroke work ↑
**CDCs**					
Sano et al. [41]	Intracoronary	3.0 × 10^5^	PAB	Rat	RV EF ↑, fibrosis ↓, inflammation ↓
Wehman et al. [42]	Intramyocardial	1.0 × 10^6^	PAB	Pig	RV FAC ↑, fibrosis ↓, RV dimension ↓

*EF* ejection fraction, *FAC* fractional area change, *PAB* pulmonary artery banding, *RVfx* RV function

**Table 2:** Clinically applied cell-based RV-centric studies in pediatric patients

	Route	Dose	Disease	Number	Outcome
**BM-derived MSCs**					
Rupp et al. [33–35]	Intracoronary	2.7 × 10^8^ (variable)	Congestive heart failure from DCM/CHD	9 case series	Modest response, EF ↑, BNP ↓
Bergmane et al. [36]	Intramyocardial	1.7–12.2 × 10^7^	DCM	7 case series	EF ↑, NT-proBNP ↓
**UCB-derived MNCs**					
Burkhart et al. [40] * NCT01883076* ^*ongoing*^	Intramyocardial	3.0 × 10^7^/kg	HLHS	10 (reported) 30 (projected) phase II trial	Preserved RV function, no adverse events
**Allogenic MSCs**					
Kaushal et al. [45] *NCT03525418* ^*ongoing*^	Intramyocardial	2.5 × 10^5^/kg	HLHS/uAVSD	30 (projected) phase I/II trial	Not published
**Cardiosphere-derived cells**					
Ishigami et al. [27] *NCT01273857*	Intracoronary	3.0 × 10^5^/kg	HLHS	14 phase I trial	EF ↑, HF ↓, growth ↑, collaterals ↓, safe, no adverse events
Ishigami et al. [44] *NCT01829750*	Intracoronary	3.0 × 10^5^/kg	HLHS	34 phase II trial	EF ↑, HF ↓, growth ↑, fibrosis ↓
JRM Co. Ltd. *NCT02781922* ^*ongoing*^	Intracoronary	3.0 × 10^5^/kg	HLHS	40 (projected) phase III trial	Not published

*BNP* brain natriuretic peptide, *CHD* congenital heart disease, *DCM* dilated cardiomyopathy, *EF* ejection fraction, *HF* heart failure, *HLHS* hypoplastic left heart syndrome, *uAVSD* unbalanced atrioventricular septal defect

A few animal studies have been performed with bone marrow-derived mesenchymal stem cells (MSCs) targeting the RV with varying results. Attenuated RV hypertrophy and RV dimensions have been described following the administration of neonatal bone marrow-derived MSCs in a mouse PAB model [31]. In one report by Wehman et al., intramyocardial delivery of human bone marrow-derived MSCs in a neonatal pig RV pressure-overload PAB model resulted in enhanced angiogenesis, an increase in endogenous levels of c-kit+ putative cardiac stem cells, increased proliferation of cardiomyocytes and endothelial cells, reduced RV hypertrophy, and improved RV function compared to controls [32]. With regard to clinical experience in pediatric populations, bone marrow-derived MSCs have been more extensively tested in children with dilated cardiomyopathy with a primary focus on reversing LV dysfunction [33, 34], but MSCs have also been tested in a limited number of HLHS patients. In one study, Rupp et al. delivered MSCs via stop-flow intracoronary injection, and the reported beneficial effects were modest and biased by simultaneous drug therapy [35]. Bergmane et al. delivered MSCs via a direct intramyocardial application and found improved ventricular function and no observed side-effects [36]. Interestingly, in this study, the authors delivered cells into the interventricular septum rather than the RV free wall.

Intramyocardial injection of cord blood-derived MSCs and mononuclear cells has been tested in small and large animal models of PAB. In mice, cord blood-derived mononuclear cells led to a reduction of RV fibrosis and increased neovascularization [37]. In an ovine PAB model, transplantation of cord blood-derived MSCs led to improved RV compliance and recruitable stroke work [38]. These studies were translated to clinical application in patients with hypoplastic left heart syndrome [39]. Investigators at the Mayo Clinic have formed a collaboration to build an HLHS consortium, currently comprised of twelve centers across North America. In their phase I clinical trial testing intramyocardial injection autologous umbilical cord blood-derived mononuclear cells, the Mayo team did not find any adverse effects of cell therapy, although no improvements in RV function were reported either [40].

Allogenic MSCs have been clinically investigated in ischemic and non-ischemic cardiomyopathy in adult patients [49]. An ongoing phase I/II trial is currently assessing their role in pediatric patients with HLHS, but results have yet to be reported [45]. In this trial, the investigators are performing intramyocardial injections at stage II palliation (the second surgical intervention of the commonly used three-step strategy to separate systemic and pulmonary circulation). Primary endpoints are focused on safety, but the investigators will also assess RV function. Another variant of MSCs, multipotent bone marrow stromal stem cells or allogeneic mesenchymal precursor cells [50], is investigated in a currently recruiting phase I/II clinical trial in patients with HLHS and unbalanced atrioventricular septal defect. These cells have not been tested in RV-specific animal models to our knowledge, but beneficial effects were hypothesized based on experience in animal models of LV failure and clinical trials in adults [51].

Another cell type that has gleaned considerable interest for application in RV failure is cardiosphere-derived cells (CDCs). CDCs are a heterogeneous population of cardiac cells derived from cardiac biopsy specimens that yield spherical clusters in culture. Working in a rat pulmonary banding model, Sano and colleagues reported that the intracoronary infusion of CDCs mediated multiple beneficial outcomes including reduced myocardial fibrosis, attenuated inflammatory markers, and improved RV ejection fraction relative to vehicle alone [41]. Wehman et al. investigated c-kit+ cells, a related cardiac resident population that can also be isolated from cardiospheres, in an animal model of RV failure. They found that intramyocardial injection of c-kit+ cells mediated improvements in RV dilatation, fractional area change, strain, and fibrosis in a swine PAB model [42]. Interestingly, this same group later reported that equivalent beneficial effects could be mediated by the delivery of the secretome of c-kit+ cells rather than the cells themselves [43]. CDCs have also been fairly extensively tested in clinical trials [27, 28, 41, 44]. The ongoing APOLLON trial is the phase III extension of the preceding phase I (TICAP) and II (PERSEUS) trials. In the latter studies, the Oh group showed that after a single intracoronary dose of CDCs 4–9 weeks after surgery, RV function improved and mortality was lower in patients with reduced ejection fraction preoperatively [41].

Because neither MSCs nor CDCs have appreciable myogenic potential [52, 53], such cell types are thought to mediate potential beneficial effects via indirect mechanisms rather than direct incorporation into the failing RV. Other candidate cell types have received less study in the context of RV-centric disease but may be capable of operating via more direct regenerative mechanisms. Human pluripotent stem cell-derived cardiomyocytes (hPSC-CMs) are a particularly promising cell source for cardiomyocyte regeneration, and their transplantation has been shown to result in the direct formation of electrically integrated new myocardium in rodent, swine, and non-human primate models of LV myocardial infarction [54–57]. Beneficial effects on LV contractile function have also been reported, including in large animal models [58, 59]. However, the use of hPSC-CMs is currently limited by their relative immaturity, low engraftment rates, incomplete electromechanical integration, and arrhythmogenic potential [55]. A number of strategies are currently being pursued to overcome these limitations including the generation of more mature cardiomyocytes, gene-editing approaches, or the application of engineered heart tissues populated by hPSC-CMs.

Although preclinical experience with hPSC-CMs has been limited to testing in models of LV-centric disease (almost exclusively myocardial infarct models), the failing pressure-stressed RV has a number of unique characteristics that make it a potentially attractive target for hPSC-based therapies at least conceptually. First, there is good reason to suppose that graft cell survival will be enhanced in this context relative to transplantation into the LV in ischemic heart disease. Mechanical washout due to high intramyocardial pressure, flushing by the coronary vasculature, leakage from the injection site, and graft cell death due to enzymatic harvesting, anoikis, ischemia, inflammation, and the fibrotic infarct environment have all been identified as contributing causes for low engraftment rates in ischemic LV myocardium [60]. Capillary rarefaction and ischemia are less pronounced in pressure-overloaded RV overload than in the LV in ischemic heart disease, and these changes occur more gradually and are diffusely distributed across the ventricle. The RV is therefore expected to be a more hospitable environment than the infarct scar or border zone, which may facilitate robust engraftment at lower cell doses. Electromechanical integration may also be enhanced in the RV, as connexin expression is commonly more downregulated in the ischemic environment and the infarct scar acts as a large barrier to host–graft physical contact. Finally, the protracted clinical course of RV failure caused by increased afterload allows for intervention early in the disease process, i.e., before fibrosis, ischemia, and inflammation have fully developed.

Despite these promising characteristics, hPSC-CM transplantation has not been previously explored in preclinical models of RV pressure overload. To our knowledge, the sole published experience with PSCs in the context of the RV was performed by Huang et al. [61]. The latter authors tested repeated intravenous injection of undifferentiated mouse induced pluripotent stem cells (miPSCs) as well as repeated intraperitoneal injection of miPSC-CMs in a rat model of monocrotaline-induced pulmonary arterial hypertension. They reported that the administration of both undifferentiated miPSCs and miPSC-CMs reduced RV hypertrophy and systolic pressure, and they attributed these effects to an anti-inflammatory mechanism. It is certainly the case that any beneficial effects must have been indirect rather than direct, given the peculiar route of administration employed in this provocative study, and it warrants replication. In our opinion, undifferentiated PSCs are an unlikely choice for cell therapies given the heightened risk of teratoma formation.

Regardless of the cell type used, the beneficial effects of stem cell transplantation have usually been attributed to indirect mechanisms (e.g., release of paracrine signals by the graft cells), rather than electromechanical integration of the graft cells and a direct contribution to force generation. This is particularly true for clinical trials which have typically involved relatively small quantities of cells, so indirect modulation of inflammation, fibrosis, and/or remodeling seems a much more likely explanation for any observed beneficial effects. Given this, there have been significant efforts in the field to recapitulate the beneficial effects of cell therapy by instead delivering cell-derived factors, including naturally secreted nanovesicles known as exosomes [62]. While the subject of much activity in LV-centric diseases, there have been very limited efforts to explore this cell-free approach in models of RV failure. For example, Bittle et al. examined the effects of human CDC-derived exosomes in a porcine PAB model and reported reduced RV hypertrophy relative to vehicle controls as well as encouraging functional effects with some but not all CDC preparations tested [63]. Clearly, more study is warranted, as experiments in models of ischemic injury in the LV suggest that cell secretome can actually outperform cell-based strategies in terms of contractile recovery in some circumstances [43]. On the other hand, it remains unknown whether the indirect actions of a cell-free therapy, perhaps in combination with myocardial hypertrophy, will suffice to sustain the RV in the face of elevated afterload longer-term or whether the addition of force-generating units will prove essential to overcome heart failure in RV pressure overload [64].

While beyond the scope of this review focused on therapeutic applications, we also look forward to the continued development of iPSC-based in vitro models of diseases related to RV failure, such as hypoplastic left heart [65, 66] or pulmonary arterial hypertension [67]. That said, progress towards cell- or engineered heart tissue-based models of RV failure will require improved methods to specifically guide the differentiation of iPSCs into right versus left ventricular myocytes, a goal that is the focus of much activity in the field.

## Conclusions

Relief of pressure overload in RV-centric disease is frequently inadequate, temporary, or not feasible. Key areas to target therapeutically are therefore myocardial contractility, mechanical stress, oxygen supply and/or demand, fibrosis, and inflammatory response. The relative impact of these individual components and their interdependence on RV ventricular function is not fully understood. Computational modeling identified reduced myocyte contractility as an important cause of ventricular dysfunction in RV pressure overload but attributed little dysfunction to myocardial fibrosis [64]. Currently applied cell-based strategies to restore RV function are largely focused on exploiting paracrine effects with little evidence of direct or indirect cardiomyocyte regeneration. They have shown improved angiogenesis, reduced cardiomyocyte loss, decreased fibrosis, and preserved or improved ventricular function. Buttressing the diseased RV by transplantation of force-generating units has yet to be accomplished but may form an attractive alternative to counteract its functional decline. Whether any of these new therapeutic concepts translate into actual clinical benefit by improving quality of life or by reducing morbidity and mortality has yet to be proven. Relevant changes in outcome with preserved or improved cardiac function will need to be demonstrated in long-term follow-up and must take into account the complex comorbidities and management strategies associated with this challenging patient population.

## Data Availability

Not applicable.

## Abbreviations

BNP: Brain natriuretic peptide
CDCs: Cardiosphere-derived cells
CHD: Congenital heart disease
CTGF: Connective tissue growth factor
DCM: Dilated cardiomyopathy
EF: Ejection fraction
FAC: Fractional area change
Glut1: Glucose transporter 1
HIF1α: Hypoxia-inducible factor 1α
HF: Heart failure
HLHS: Hypoplastic left heart syndrome
hPSC: Human pluripotent stem cells
hPSC-CM: Human pluripotent stem cell-derived cardiomyocyte
iPSC: Induced pluripotent stem cell
FOXO1: Forkhead box protein O1
LV: Left ventricle
MEF-2: Myocyte enhancer factor-2
MHC: Myosin heavy chain
miPSC: Mouse induced pluripotent stem cell
MMP: Matrix metalloproteinase
MSCs: Mesenchymal stem cells
PAB: Pulmonary artery banding
PDK: Pyruvate dehydrogenase kinase
PSCs: Pluripotent stem cells
PSC-CMs: Pluripotent stem cell cardiomyocytes
ROS: Reactive oxygen species
RV: Right ventricle
RVfx: RV function
RyR2: Ryanodine receptor 2
SERCA: Sarcoplasmic reticulum Ca^2+^-ATPase
Sp1: Specificity protein 1
TEF-1: Transcriptional enhancer factor 1
TIMP: Tissue inhibitor metalloproteinase
TGF-β1: Transforming growth factor β1
uAVSD: Unbalanced atrioventricular septal defect
VEGF: Vascular endothelial growth factor
